# GENOME-WIDE RELAXATION OF SELECTIVE CONSTRAINTS UNDERLIES THE EVOLUTION OF SHORT LIFE SPAN IN AFRICAN KILLIFISHES

**DOI:** 10.1093/geroni/igz038.027

**Published:** 2019-11-08

**Authors:** Dario Riccardo Valenzano, Rongfeng Cui

**Affiliations:** 1 Max Planck Institute for Biology of Ageing, Cologne, Germany; 2 Max Planck Institute for Biology of Ageing, Cologne, Nordrhein-Westfalen, Germany

## Abstract

African killifishes independently evolved annual life cycles at least three times, offering a unique natural experiment of diversification of life history strategies. Using a comprehensive whole-genome sampling of 46 species of African killifishes, we found that genome size correlates with annual life style and climate. Annual species underwent genome-wide expansion of transposable elements, higher gene family turn-over rates and relaxed selection in genes in known aging pathways, such as mitochondrial replication and translation, mTOR pathway and DNA repair. Whole-genome resequencing in wild Nothobranchius populations showed bottle-necks and a genome-wide signature of relaxation of selection in populations evolved in dryer climates. In conclusion, evolution in ephemeral environments in African killifishes caused an extensive relaxation of selective constraints at genome-wide level. We discovered that, in African killifishes, ecology drove the evolution of short life span, associated to tens of thousands of slightly deleterious mutations driven to intermediate to high frequencies.

